# Patterns and obstacles to oral antidiabetic medications adherence among type 2 diabetics in Ismailia, Egypt: a cross section study

**DOI:** 10.11604/pamj.2015.20.177.4025

**Published:** 2015-02-25

**Authors:** Khaled Heissam, Zeinab Abuamer, Nahed El-Dahshan

**Affiliations:** 1 Family Medicine Department, Faculty of Medicine, Suez Canal University, Ismailia, Egypt

**Keywords:** Adherence, antidiabetic medication, type 2 diabetes, self-management

## Abstract

**Introduction:**

Diabetes is a costly and increasingly common chronic disease. Effective management of diabetes to achieve glycemic control improves patient quality of life. Adherence rates to drug regimens in patients with type 2 diabetes are relatively low and vary widely between populations. There are many factors that could affect patient adherence to drug therapy. The aim of the present study was assessing patterns and obstacles to adherence of type 2 diabetic patients to their oral hypoglycemic drugs.

**Methods:**

The present work is a descriptive cross section study, carried on type 2 diabetic patients who were on oral hypoglycemic drugs. Data concerning adherence to drugs was assessed using measure treatment adherence scale (MTA).

**Results:**

A total of 372 (55.59% males and 44.41% females) patients with type-2 diabetes fulfilled the inclusion criteria and included in the study. Among the participants, 26.1% were found to have good adherence, 47.9% had a fair adherence, and 26% had poor adherence.

**Conclusion:**

The overall rate of medication adherence among the diabetic patients population was suboptimal and non-acceptable. Evaluation of adherence is vital for patients with diabetes in order to determine factors and barriers affecting the adherence and to manage them.

## Introduction

Diabetes is a costly and increasingly common chronic disease. The data from the Egyptian Demographic and Health Survey in 2008 showed the crude prevalence rate of physician-diagnosed diabetes among the adult population of Egypt aged 15-59 to be 4.07% [1]. Despite the clinical and economic value of the glycemic control is clear, many diabetic patientsstilldevelopdiabetes related complications, hence increases the burden on patients and the health services. Achievement of optimal glycemic control reduces the likelihood of diabetic complications and risk of death. However, achieving blood glucose level as close to normal as possible relays on the rational use of available anti-diabetic regimen, good adherence to prescribed treatments and successful self-management by patients [2]. One of the reasonsbehind unachievable glycemic controlmay be due to lack of patient's adherent to therapeutic regimen. Adherence rates to drug regimens in patients with type 2 diabetes are relatively low and vary widely between populations. It is estimated that only third of diabetic patients have adequate treatment adherence [3]. Physicians and nurses can motivate patients to be more adherentto their anti-diabetic regimen if they work on the factors that possibly affect oral hypoglycemic medications adherence. Many factors cancontribute and affect patient adherence to drug therapy. However, physicians and nurses can motivate patients to be more adherences to their anti-diabetic regimen and work on the causes. The main factors can be divided into three groups: patient factors, social and medical support, and medication related aspects [4]. Patient factors are for example the patient's age (i.e. older patients are more adherent), economic status (patients with a higher economic status being more adherent) and health beliefs (patients with beliefs about medicines as something harmful are less adherent) [4, 5]. Social and medical support included among others family help and the patient-health care provider relationship; patients with more support are more adherence. Medication related factors take into account the attitude towards medicines, the complexity of the medication regimen and the experience of side effects [6]. A positive attitude towards medicines, a less complex medication regimen and less experience of side effects are related to higher adherence rates. Studies that focused on the patient's perspective and his/her experiences with drug adherence have been performed less frequently [7]. Few studies have investigatedthe adherence to oral hypoglycemic medication in our Egyptian society, which has special demographic characteristics in urban and rural areas. Thesestudies have different designs and inconsistent results, leading to difficulties ingeneralizing their results on our diabetic population. Therefore, the aim of the present study was toassess patterns and obstacles that affecting adherence in type 2 diabetic patients to their oral hypoglycemic drugs.

## Methods

An ethical approval was obtained from the research and Ethical Committee of the Faculty of Medicine, Suez Canal University. The objectives of the study were explained to individual patients and voluntary informed consent of the patients was also taken. The study was conducted in the Fanara Family Medicine Center that belongs to the Faculty of Medicine, Suez Canal University. Fanara is a rural areathat located 45 Km south Ismailia governorate, Egypt. The data was collected over the period between the beginning of April and end of May 2013. Study Design:The present study is a descriptive cross section study that was carried on all type 2 diabetic patients who lived in Fanara city andhave medical records at Fanara's Family Medicine Center. Exclusion criteria werepatients who were on insulin therapy, unconscious, or attended emergency condition and, or who were not interested in the study. A qualitative structured questionnaire was first used as a pilot in this study, and was testedon 40 type 2 diabetic patients. These patients were subsequently excluded from the study. After the pilot testing, some question-items in the questionnaire were modified and reframed to ensure validity of the method. The 36-item questionnaire took an average of 20 minutes to fill in and was administered to the participantsat the study site. The questionnaire was designed to have two sections; the first section included the socio-demographic characteristics of the type 2 diabetic patients while the second section assessed the adherence to oral anti-diabetic drugs. Each sectionconsisted of opened-/closed-ended questions. The adherence section focused on exploring patients’ experience with current anti-diabetic prescriptions and possible factors that could affect the adherence and patients’ knowledge and practice of diabetes self management behaviors such as self blood glucose monitoring, optimal blood glucose target, and complications from poor glycemic control. Data concerning adherence to drugs, its effect and its determinants, was assessed using questions of the Measure Treatment Adherence scale developed by Delgado and Lima. This method is used frequently to measure patient compliance with drug treatment. The Measure Treatment Adherence scale is a variation of the Morisky-Green test, which was used to assess patient behavior patterns associated with the use of medicines. It also showed good concurrent validity with high correlations with any answer and 0.77 sensitivity and 0.73 specificity [8]. All questions were read out to the participant, and the answers were recorded. Patients achieving a result of more than 75% were included in the good compliance group. Patients achieving a result less than 50% were included in the poor compliance group. Patients achieving a result between 50 and 75% were included in the fair compliance group. All collected data were analyzed using the Statistical Package for the Social Sciences (SPSS), version 13 software. Tests of proportions were done with ANOVA and a p-value of

## Results

A total of 372 males (209 (55.59%) and 167 females (44.41%))patients with type-2 diabetes who fulfilled the inclusion criteria were included in the study. The mean age was 51.64±10.76 years. The sociodemographic characteristics of the studied population showed that among the patients, 159 (42.29%) had no formal education,100(26.60%) can read and write, 61 (16.22%) had received high education and 56 (14.89%) had received basic education. Also, 221 (58.78%) of the patientswere married, 109 (28.99%) were widows, 40 (10.64%) were divorced and 6 (1.6%) were single. A total of 144 (38.3%) were unemployed and 228 (61.7%) had a job. Among the participants, 256 (68.09%) perceived their economic standard as being below their needs and 120 (31.91%) perceived it as being enough or more than their needs. In addition, 217 (57.71%) had a family support and 159 (42.29%) had no family support. The results from the adherence section are summarized in Table 1. Among the patients, 98 (26.1%) were found to have good adherence, whereas 180(47.9%) had a fair adherence, and 99(26%) had poor adherence. Statistical analysis of factors that could affect adherence to oral hypoglycemic drugs results is illustrated in Table 2. Most of the patients (255 (67.82%)) had weak believes and motivations. In addition, the majority of the participants (230 (61.17%)) had poor relationship with health care providers. Another affecting factor is monitoring their blood glucose level. Theresults found that 272 (72.34%) participants did not monitor their blood level regularly. In addition, the number of drugs taken was found to be another factor, the present study found that 134 (35.64%) patients had only one oral hypoglycemic drug, and 242 (64.36%) had more than one oral hypoglycemic drugs. Among the patients, 246 (65.43%)had experienced a side effect of their oral hypoglycemic drug. On the other hand, 258 (68.62%) patients could not get their oral hypoglycemic drugs regularly because of its direct and indirect cost in relation to their income. Finally, 196 (52.13%) of allpatients had poor knowledge about diabetes and its complications. The ANOVA test using post Hoc for multi comparisons analysis through all the studied factors and different adherence scale compare frequency of these factors that could affecting adherence to oral hypoglycemic drug (OHD) among different adherence scale groups found highly significant different at P Table 3. The results showed that 201(53.46%) of the participants sometimes have forgotten to take medications, and 33 (8.78%) of the participants always forgotten to take medications. Of all the participants 187(49.73%) sometimes careless to take medications at time while 31(8.24%) participants were always careless to take medications at time. On the same times 162(43.09%) of the participants sometimes stopped taking medications when becomewell. On the other hand, 30 (7.98%) of the participants always stopped taking them. Also, 214(56.91%) of the participants sometimes stopped taking medications when become worse, but 29(7.71%) of theparticipants always stopped taking them. In the same manner, 168(44.68%) of the participants sometimes hate pills while 30(7.98%) participants always hated them. Regarding the medical advisory, 201(53.46%) of the participants sometimes stopped it and 30(7.98%) of the participants always stopped it. Table 4 shows the linear regression analysis model to assess the association between adherence to oral hypoglycemic drugs and selected independent adherence factors. There was statistical significant effect of the patient and healthcare provider′s relationship as P value was 0.046 and beta was 0.186 (CI95% (-0.547-0.005)). Thus, the only factor that could be used as potential predictors of adherence to oral hypoglycemic drugs was patient healthcare providers relationship.

**Table 1:** Frequency of overall MTA scale to oral hypoglycemic drugs among the participants

Adherence level	No (%)
Good	98 (26.1)
Fair	180(47.9)
Poor	99 (26)
Total	376 (100)

**Table 2:** Comparing the frequency of the factors affecting oral hypoglycemic drug adherence among different adherence scale in study population (n = 376)

Factors		Adherence scale			No (%)	P value
		Non	Fair	Good		
**Patient believes and motivation**	Good	6	64	51	121(32.18)	0.000
	Weak	93	115	47	255(67.82)	
**Patient healthcare providers relationship**	Good	9	79	58	146(38.83)	0.000
	Poor	90	100	40	230(61.17)	
**Monitoring blood glucose level**	Regular	5	54	45	104 (27.66)	0.000
	Irregular	94	125	53	272(72.34)	
**Numbers of drugs taken**	Monotherapy	13	70	51	134(35.64)	0.000
	Combination	86	109	22	242(64.36)	
**Drug regimen**	Complex	79	99	41	219 (58.24)	0.000
	Simple	20	80	57	157(41.76)	
**Experience side effect**	Present	84	111	51	246(65.43)	0.000
	Absent	15	68	47	130(34.57)	
**Direct, indirect cost income**	Adequate	19	63	36	118(31.38)	0.009
	Not adequate	80	116	62	258(68.62)	
**Patient knowledge**	Rich	7	53	40	101(26.86)	0.000
	Adequate	16	38	25	79(21.01)	
	Poor	76	88	33	196(52.13)	
**Total**		99	179	98	376(100)	-

**Table 3:** Assessment of patient adherence to oral hypoglycemic drug using the MTA Scale (n = 376)

MAT scale items		Frequency	Percent
Forgotten to take medications	Always	33	8.78
	Usually	57	15.2
	Sometimes	201	53.5
	Never	85	22.6
Careless at time	Always	31	8.24
	Usually	101	26.9
	Sometimes	187	49.7
	Never	57	15.2
Stop taking medication when better	Always	30	7.98
	Usually	147	39.1
	Sometimes	162	43.1
	Never	37	9.84
Stop medication when worse	Always	29	7.71
	Usually	49	13
	Sometimes	214	56.9
	Never	84	22.3
Hate pills	Always	30	7.98
	Usually	145	38.6
	Sometimes	168	44.7
	Never	33	8.78
Stop medical advisory	Always	30	7.98
	Usually	83	22.1
	Sometimes	201	53.5
	Never	62	16.5

**Table 4:** Linear regression analysis model to assess the association between oral hypoglycemic drug adherence and selected independent adherence barriers (n = 376)

	Unstandardized Coefficients	Sth. Error	Standardized Coefficients	T	Sig.	95% Confidence Interval for B	
						Lower Bound	Upper Bound
(Constant)	1.77	0.258		6.856	0.000*	1.262	2.278
Patient believes and motivation	-0.108	0.152	-0.07	-0.711	0.478	-0.406	0.191
Patient healthcare providers relationship	-0.276	0.138	-0.186	-2.004	0.046*	-0.547	-0.005
Monitoring blood glucose level	-0.184	0.107	-0.114	-1.711	0.088	-0.395	0.027
Drug regimen	0.088	0.138	0.06	0.639	0.523	-0.184	0.36
Experience side effect	0.121	0.136	0.079	0.886	0.376	-0.147	0.389
Direct, indirect cost income	-0.07	0.077	-0.045	-0.905	0.366	-0.222	0.082

## Discussion

Diabetes mellitus is a chronic disease that requires continuing medical care and education to prevent acute complications and reduce the risk of long term complications. Thus, there is a need for an integrated approach to facilitate adherence that addresses patient′s motivation to follow treatment advice as well as their ability to do so [9]. The study was carried out aiming to: assess patterns and obstacles of oral hypoglycemic drugs adherence among type 2 diabetic patients to reach recommendations to improve the patient's adherence. The results of the present study show that, among the participants 26.1% were found to have good adherence, 47.9% had a fair adherence, and 26% had poor adherence. These results are not in agreement with Nahla et al., [10], who reported that about 57% of patients always took their medication as prescribed and on time. In addition, the results by Kravitzetet al., 11 in Scotland found that 91% of the diabetic patients reported that they actually took their medication as prescribed. In the same manner, our results are inconsistent with the results of Gimenes and his colleges who found that the patient's adherence level to antidiabetic therapy was 78.3% among their study sample [12]. Finally, Yelena et al., in 2008 proved that the overall oral antidiabetic medication was 81% [13]. This difference in results may return to awarenessdifferences in the importance of adherence to antidiabetic medication and the policies and strategies that different countries adopt. Moreover, we found that 67.82% of the participants had week believes and motivations about the disease with statistical significant effect on adherence. These findings are in agreement with Spikmans et al.,^14^. Also, the results of the present study was in agreement with results of Donnan et al., [15] and Nagwa [16] who showed that the majority of patients had a strong perception about seriousness of the disease and the benefits of adherence to treatment with significance effect. This is an important point that physician should understand culture, perceptions and believes of their patients before recommending the treatment. Also, 61.17% of the participants had poor communications with health care providers with statistical significant among different adherence scale. The resultsare in agreement with Rolla [17] and Rubin [18], and in contrast to Shams and Barkat [19] who showed that the majority of patients had good communications with health care providers with no statistical significance on adherence level.

The present study showed that (72.34%) were not monitoring their blood sugar regularly with statistical significant effect on adherence. Many of our diabetic patients were not aware of self-monitoring of blood glucose level at home (SMBG) or lack financial support to buy the apparatus for regular and prompt detection of fluctuations in their blood glucose levels. This finding was in conformity with the report of a study made by Harris et al. [20] in US where many diabetic patients reported never to have monitored their blood glucose levels. The absence of established guidelines on SMBG and lack of its perceived importance by patients, as well as, the cost of the blood glucose monitoring devices especially in a developing country as in Egypt may have accounted for the low level of regular blood sugar monitoring among patients. Concerning medicationrelated factors: Our study revealed that patients who take complex treatment more than who take simple treatment with significant effect on adherence rate and this indicate that once daily dosing is the best. This disagrees with Iskedjian et al. [21], Shams and Barakat. [19], Sweileh et al [22] who showed that the non-adherence was least with the single drug regimen while it was highest among patients who were on combined oral and insulin treatment. The current study showed that 65.43% had side effects towards the treatment with statistical significant effect on adherence scale. This was in agreement with the findings in the study made by Jayant et al. [23] who reported that the side effects of medication may be a significant factor that can affect diabetic patients’ long-term adherence to treatment programs and this was a main factor for limiting adherence. Another study made by Girered [24] who reported something different than our results as he found that majority of diabetic patients (58%) had side effects with no statistical significant effect on adherence. Moreover, the present study showed that 68.62% of the patients had not adequate cost of medications in relation to the income with statistical significant effect on adherence. The same results was conducted with Shams and Barakat [19] who showed the same results where is a significant higher rate of adherence to oral treatment was observed in patients who exhibited adequate healthcare costs in relation to their income or full coverage health insurance compared with the others who did not have. This was also in agreement with Ohene-Buabeng et al., [25] and Adisa et al., [26] who reported that financial variables especially the direct and indirect costs associated with a prescribed regimen and restricted access to therapy had been found by several studies to influence patients’ commitment to medication adherence in developing countries and patients who had no insurance coverage or who had low income were more likely to be non-adherent to treatment In regards to patient-related factors,52.13% had poor knowledge about the disease and there was a strong positive relation between total knowledge and adherence rate to the medication. Fitzgerald et al., [27] found that the level of adherence increased with the improvement of the patient's level of knowledge. On the other hand, findings of the study conducted in China by Chan et al., [28] indicated that there was no association between the patient′s knowledge and adherence.

The current study also noted that only 26.1% had good adherence and 47.9% had fair adherence towards treatment. This was in agreement with Wild [29] who reported that 39% of patients had good medication adherence, 49% medium adherence and 12% poor adherence. These results are not in agreement with Nahla et al., [10] who reported that about 57% of patients always took their medication as prescribed and on time. Also results of the study by Kravitzet al., [11] in Scotland found that 91% of the diabetic patients reported that they actually took their medication as prescribed and this difference in results may be due to differences in awareness of importance of adherence and be may return to the national health policies. Previous studies return the causes behind poor adherence to financial, being busy with work, too many medicines being prescribed. Concerning the linear regression analysis model which assessed the association between adherence and selected independent adherence barriers, the healthcare provider′s relationship was the dominant predictor to good adherence. Many other factors which could affect adherence still remain unidentified. The results of this study emphasize the considerable role of good report with the doctor and the entire treatment team for shaping the awareness of the disease and acquiring the necessary knowledge. From our results, we endorse the role of family physician to screen patients at high risk for poor adherence, and the family physician should try more than usual to apply multiple interventions in order to improve adherence including educational, behavioral, and affective interventions. Educational interventions seek to improve adherence by providing information and/or skills. Education may take the form of individual instruction or group classes. In any event, a key element of successful educational strategies is providing simple and clear messages, hopefully tailored to the needs of the individual, and verifying that the messages have been understood [29]. Behavioral approaches have their roots in cognitive-behavioral psychology and use techniques such as reminders, memory aids, synchronizing therapeutic activities with routine life events (e.g., taking pills before you shower), goal-setting, self-monitoring, contracting, skill-building, and rewards. What is important is that the behavior in question has been negotiated with and accepted by individual patients so that adoption of the behavior has a chance of succeeding in the long term. Affective interventions seek to enhance adherence by providing emotional support and encouragement. Finally, it should be remembered that application of multiple interventions of different types is more effective than any single intervention [30]. Our results should be viewed with consideration of some limitations. One limitation was the use of self-report data on medication adherence, because of a resulting tendency to overestimate adherence due to recall biases and social desirability.

## Conclusion

The overall rate of medication adherence among the diabetic patients population was found to be suboptimal and non-acceptable. Evaluation of adherence is vital for patients with diabetes in order to determine factors and barriers affecting adherence. In addition, in order to manage poor adherence and better identification of affecting factors individualized suitable recommendations are essential for better healthcare management.
